# Bone morphogenetic proteins (BMPs) at the forefront of ocular diseases and therapeutics

**DOI:** 10.1186/s40662-025-00445-1

**Published:** 2025-07-23

**Authors:** Yurong Shi, Ju Zhang, Wenxuan Duan, Linghan Gao, Yang Liu

**Affiliations:** 1 The First School of Clinical Medicine, Lanzhou University, Lanzhou, Gansu China; 2 Department of Ophthalmology, The First Hospital of Lanzhou University, Lanzhou, 730000 China

**Keywords:** Bone morphogenetic proteins, Ocular diseases, Treatment, In vivo, In vitro

## Abstract

Bone morphogenetic proteins (BMPs), belonging to the transforming growth factor β (TGF-β) family, are multifunctional growth factors predominantly distributed in human bone tissue. Some studies also have revealed that BMPs are widely expressed in ocular tissues. Over the past two decades, research on the therapeutic application of BMPs has yielded significant advancements not only in the treatment of skeletal, cardiac, renal and neurological diseases but also in ocular conditions. Both in vivo and in vitro experiments have demonstrated the significant therapeutic efficacy of BMPs in various ocular disorders, including myopia, corneal opacity, cataract, uveal melanoma, retinal detachment and other eye diseases. Studies have further identified that BMPs exert their actions through mechanisms closely associated with the canonical Smad pathway. Compared to traditional therapeutic drugs, BMPs exhibit some advantages, including low toxicity, minimal side effects, amongst others. However, numerous unresolved issues persist during in vivo and in vitro experiments. The objective of this review is to explore the advancements in the application of BMPs for the treatment of ocular diseases in animal models or in vitro experiments, and to provide some insights into the challenges that need to be addressed for the translation of BMP-based therapies into clinical practice.

## Background

Bone morphogenetic proteins (BMPs) are extracted from the bone and can induce cartilage and bone formation [1, 2]. They are pleiotropic growth factors classified within the transforming growth factor β (TGF-β) superfamily, representing the largest subfamily of the superfamily [1–3]. BMPs are not only important factors in the induction of bone and cartilage but also participate in embryonic development and adult body maintenance [4]. They play important roles in the growth, proliferation, and migration of various cell types, and are essential for the development of the heart, kidney, central nervous system, liver and lung, amongst others [3, 5–8].

**Fig. 1:**
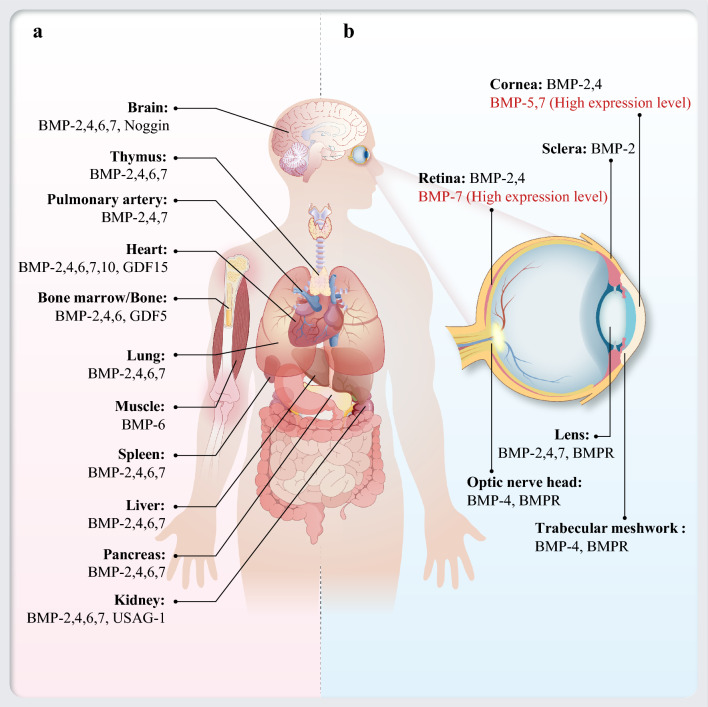
Schematic representation of the distribution of bone morphogenetic proteins (BMPs) in the body and ocular tissues. **a** The distribution of BMPs in various organs. **b** The distribution of BMPs and their receptors in ocular tissues. BMPR, bone morphogenetic protein receptor

Besides the life-sustaining organs, some BMP family members are also distributed in adult and embryonic ocular tissues, such as the cornea, conjunctiva, ciliary body, iris, lens and retina [9–13]. According to embryonic and mature animal models, the expression of BMP subtypes (BMP-2, 3, 4, 5, 6 and 7) along with three BMP receptors (BMPR-IA, BMPR-IB, and BMPR-II) has been determined in all ocular tissues, but the level of the expression in adult ocular tissues varies [11]. Previous studies have demonstrated high levels of BMP-5 and BMP-7, but low expression of BMP-2 and BMP-4 in the cornea [14]. Both trabecular meshwork (TM) cells and optic nerve head (ONH) cells can secrete BMPs, and they also express the three BMP receptors [15]. BMP-2, 4 and 7 are observed in the retina, but BMP-7 is uniquely distributed across all retinal layers, achieving the highest level in both the inner and outer nuclear layer [16, 17]. BMP-2, 4, 7 and the three receptors are all expressed in the lens cells, including the fiber cells and the epithelial cells [13, 18] (Fig. 1). Given the widespread distribution of BMPs in embryonic ocular tissues, it has been conclusively demonstrated that BMPs are intimately linked to ocular development [9–18].

Studies mainly focus on the embryonic development of the lens and the retina [19, 20]. Some studies have shown that BMPs promote lens regeneration and induce lens fiber differentiation, and found that the absence of BMPs could impede lens development [20]. Besides lens development, BMPs exhibit a strong association with the development of the retinal pigment epithelium (RPE) and the proliferation of optic cup cells [19]. Furthermore, BMPs partly determine the eye’s size and shape [21–23]. Noggin (an antagonist of BMPs) also plays an important role in the shape and size of the eye and the development of ocular tissues, such as the ciliary body and retina [24–27].

Emerging evidence from developmental studies implicates BMPs as key mediators in ocular pathogenesis. Their well-documented roles in eye morphogenesis and cellular differentiation suggest that dysregulated BMP signaling may contribute to various ocular disorders. This pathogenic involvement positions BMP pathways as promising molecular targets for therapeutic intervention in eye diseases [19–23].

**Fig. 2:**
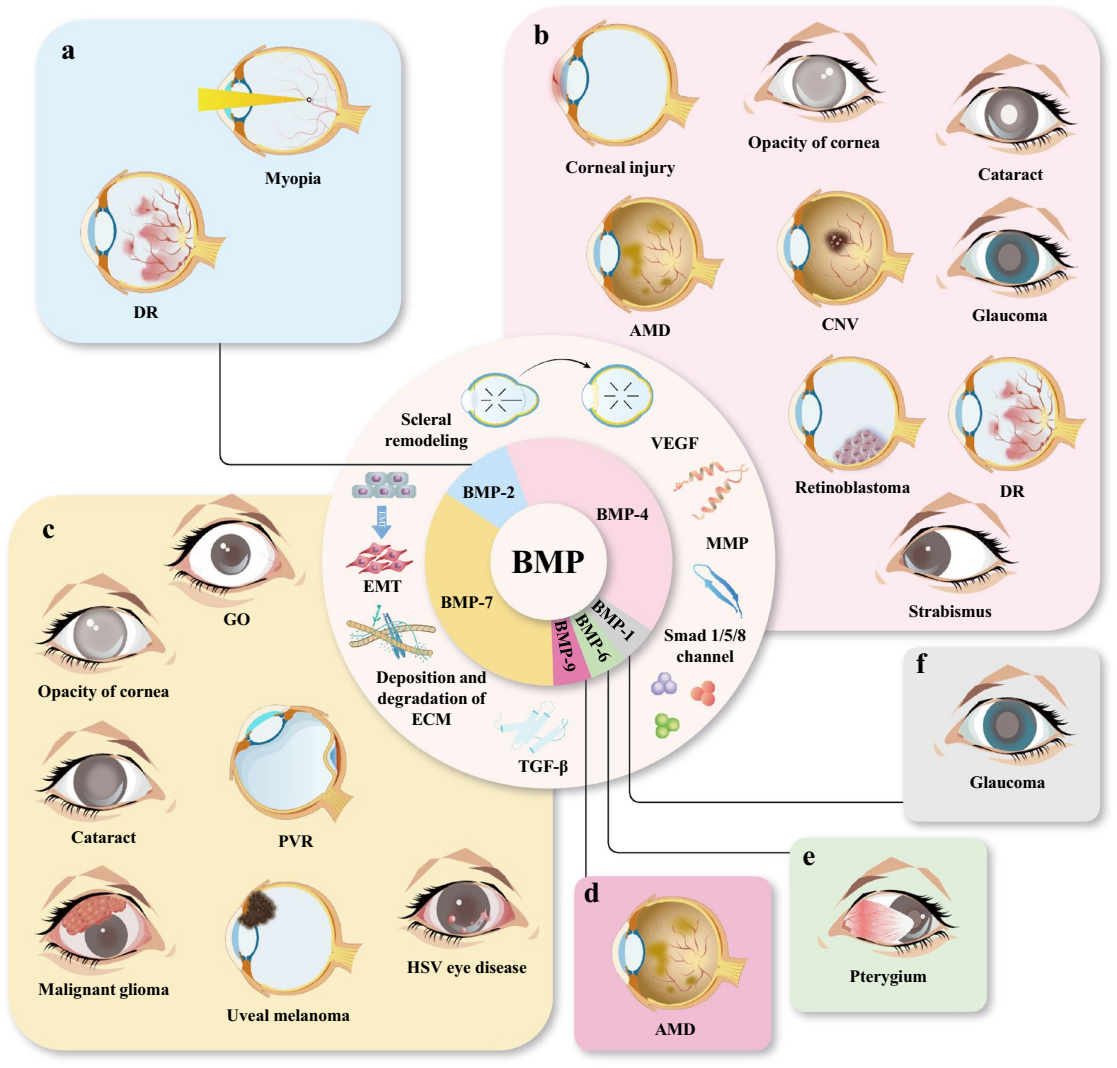
Schematic diagram of the application of bone morphogenetic proteins (BMPs) in ocular diseases and related molecular mechanisms. This schematic diagram mainly shows the BMP subtypes (including BMP-1, BMP-2, BMP-4, BMP-6, BMP-7, and BMP-9) involved in ocular diseases. **a** BMP-2 is involved in the development of myopia and diabetic retinopathy (DR) mainly by affecting scleral remodeling and the blood-retinal barrier (BRB), respectively. **b** BMP-4 participates in the pathogenesis of corneal injury, corneal opacity, cataract, age-related macular degeneration (AMD), choroidal neovascularization (CNV), glaucoma, DR and retinoblastoma through regulating the epithelial-mesenchymal transition (EMT) process and by influencing Smad signaling molecules (Smad1/5/8), vascular endothelial growth factor (VEGF), matrix metalloproteinases (MMPs) and extracellular matrix (ECM) proteins. **c** BMP-7 delays the progression of Graves’ ophthalmopathy (GO), corneal opacity, cataract, proliferative vitreoretinopathy (PVR), malignant glioma, uveal melanoma, and herpes simplex keratitis (HSV) ocular diseases through inhibiting EMT via the Smad signaling pathway, by suppressing pro-inflammatory cells and pro-inflammatory signaling molecules. **d** BMP-9 can delay AMD progression by inhibiting neovascularization. **e** BMP-6 affects the progression of pterygium by inhibiting corneal epithelial differentiation. **f** BMP-1 may be a potential biomarker of glaucoma through bioinformatics analysis. TGF-β, transforming growth factor β

This review synthesizes current knowledge of BMPs as dual regulators of ocular development and disease pathogenesis. Focusing on preclinical evidence from animal models and cellular studies, we appraise BMP-based therapeutic strategies for diverse ocular disorders including myopia, strabismus, corneal opacity, glaucoma, cataract, diabetic retinopathy (DR), and fundus diseases (Fig. 2). Furthermore, we critically analyze the translational challenges hindering clinical implementation of BMP-targeted therapies, providing a roadmap for future research directions.

## BMPs and ocular diseases

### BMPs and myopia

Myopia is the most common refractive error and the leading cause of reversible visual impairment and blindness worldwide. In recent years, the incidence of myopia has continued to rise. It is estimated that the total number of people with myopia worldwide will reach an estimated 4.8 billion by 2050, and high myopia will account for 9.8% of the worldwide population (about 938 million) [28–30]. High myopia significantly increases the risk of complications, such as myopic macular degeneration, retinal detachment, choroidal neovascularization (CNV), vitreous disease, and glaucoma, among others [28]. Therefore, investigating the pathogenesis of myopia is crucial for early intervention.

**Fig. 3:**
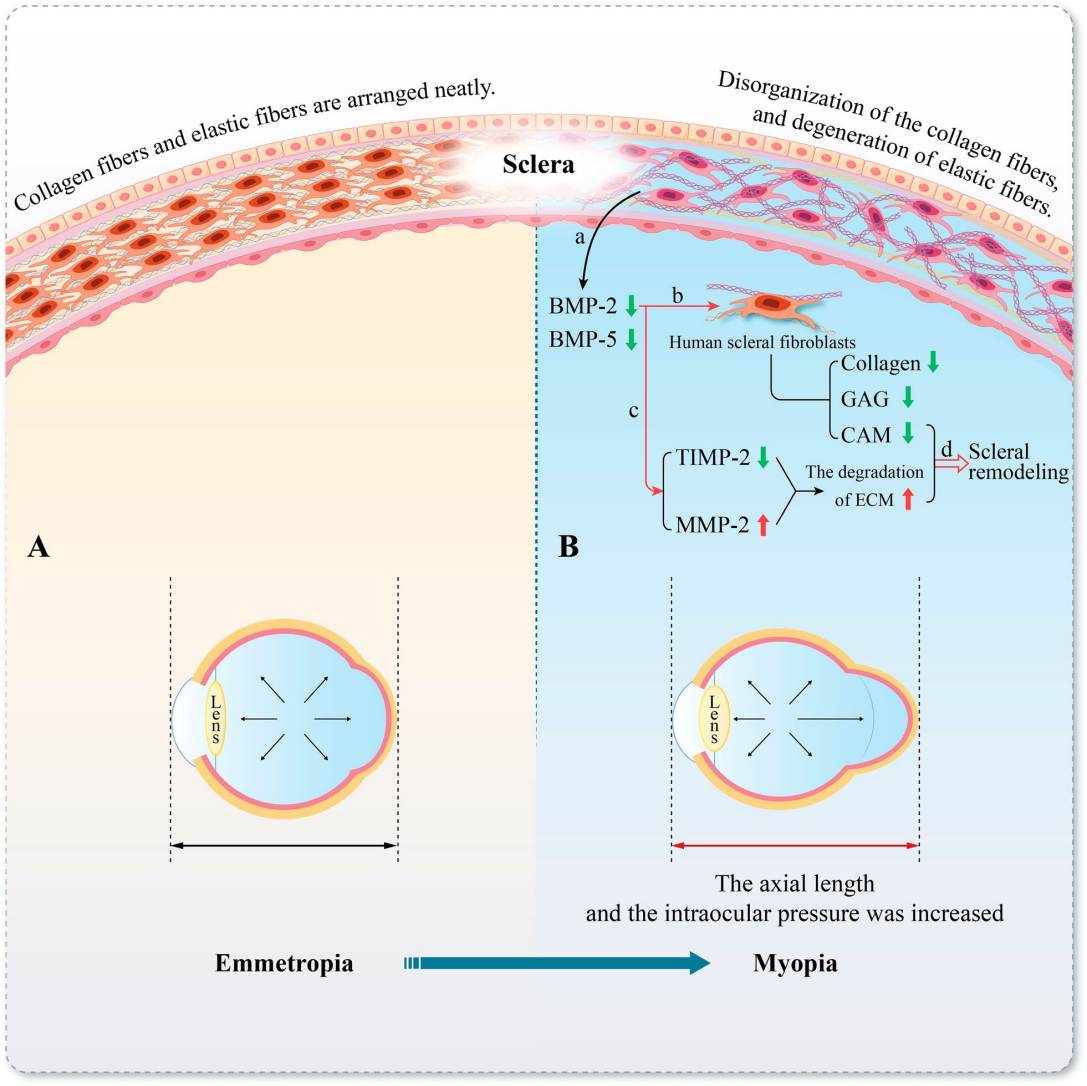
Bone morphogenetic protein (BMP)-2 and 5 are involved in scleral remodeling during myopia formation. **A** The collagen and elastic fibers are arranged neatly in the sclera tissue of the emmetropia eyes, and the ocular axis and sclera thickness are normal. **B** In the scleral tissue of myopic patients, there is a disordered arrangement of collagen fibers and degeneration of elastic fibers. The axial length of the eye increases and the posterior pole of the sclera thins, resulting in the formation of posterior scleral staphyloma. a: Compared to normal eyes, the expression levels of BMP-2 and BMP-5 are decreased in myopic eyes. b: The decreased expression of BMP-2 down-regulates the expression of type I and III collagen in human scleral fibroblasts (HSFs) and reduces the synthesis of glycosaminoglycans (GAGs) and cartilage-associated matrix (CAM). c: The decrease in the expression of BMP-2 down-regulates the level of tissue metalloproteinase-inhibitor-2 (TIMP-2), and up-regulates the expression level of matrix metalloproteinase-2 (MMP-2), thereby promoting the degradation of extracellular matrix. d: The effects of decreased BMP-2 described in b and c ultimately accelerate scleral remodeling

The progression of myopia is closely related to scleral remodeling [31, 32]. The sclera is composed primarily of collagen and elastic fibers, which plays a role in maintaining the shape of the eye and protecting intraocular tissues [33]. During myopia development, the sclera undergoes a series of pathological changes with the lengthening of the eye axis, including disorganization of the collagen fiber, and degeneration of elastic fibers and extracellular matrix (ECM) [32, 34]. Scleral remodeling causes the fibers to lose their ability to maintain the shape of the eye, which subsequently leads to the continuous elongation of the ocular axis and thinning of the posterior pole of the sclera, resulting in posterior scleral staphyloma [31, 35]. Posterior scleral staphyloma is considered to be directly or indirectly associated with myopic maculopathy, myopia-related retinal atrophy, and glaucoma [31, 36] (Fig. 3).

Existing evidence has demonstrated the association between BMPs and the development of myopia, with their involvement in the molecular mechanisms underlying scleral remodeling [37–40]. The regulatory process of scleral remodeling is mediated by multiple growth factors and proteolytic enzymes, including TGF-β, BMPs, and matrix metalloproteinase-2 (MMP-2) [32]. Accumulated research findings consistently identify BMP family members and their cognate receptors in human scleral specimens. BMP-2, 4, 5, and 7 have been found in the sclera, with BMP-5 having the highest level of expression, followed by BMP-2, BMP-4, and BMP-7 [37]. In addition to the findings in the human sclera, some studies further explored the expression of BMPs and their changes in the sclera of the myopic guinea pig model induced by form deprivation. The results confirmed that the expression and level of BMPs in the sclera varied with the development of myopia [37–40]. By comparing the myopic eyes and the control eyes of the guinea pigs, the expression of BMP-2 and BMP-5 decreased in the myopic eyes, suggesting that they were associated with scleral remodeling during the myopia induction [37, 39]. Zeng et al. also found that the expression of BMP-2 decreased in the retina of guinea pig myopic eyes [38, 40]. Based on these studies, BMP-2 and BMP-5 are assumed to be involved in the development of myopia and their expression is seen to decrease during this process (Fig. 3). Moreover, other studies verified the effects of BMP-2 on human scleral fibroblasts (HSFs) which is crucial for scleral remodeling. BMP-2 could stimulate the mitosis of HSF DNA and promote the proliferation of HSF cells. After examining the expression levels of Smad1/5/8 signaling molecules, the researchers found that the classical Smad signaling pathway might be activated in HSFs in response to BMP-2 stimulation [37]. Some experiments also explored the effect of BMP-2 on ECM synthesis in HSFs cultured in vitro. They found that BMP-2 increased the expression of collagen types I and III and promoted the synthesis of glycosaminoglycans (GAGs) and cartilage-associated matrix (CAM) in HSFs [38].

Matrix metalloproteinases (MMPs) are also involved in the degradation of ECM fibers, and the balance between activation and inhibition of MMPs is critical for scleral remodeling [32]. Studies have confirmed that BMP-2 can reduce MMP-2 expression and promote tissue inhibitor of metalloproteinases-2 (TIMP-2) expression in HSFs [41] (Fig. 3). Based on the effects mentioned above, BMP-2 can enhance collagen and GAGs synthesis to increase the stiffness of the sclera and slow axial lengthening. On the other hand, BMP-2 can elevate the level of TIMP-2 to diminish MMP-2 activity of degradation for collagen and GAGs, thereby delaying the progression of myopia [41]. Thus, BMP-2 has the potential to assist in delaying the progression of scleral remodeling [37–41].

Although the human sclera does not contain cartilage, it still retains the potential for cartilage formation during the evolutionary process [42]. While the role of BMP-dependent scleral remodeling in myopia remains to be fully characterized, BMP-2’s known induction of cartilaginous ECM proteins suggests a potential dual mechanism: stabilizing scleral structure against extraocular muscle (EOM) forces and influencing accommodation responses, which may retard axial elongation [38]. This hypothesis requires further investigation into the effects of BMP-2 and BMP-5 on changes in scleral proteoglycans and collagen synthesis, which may help to understand their roles in scleral remodeling.

Besides the effects of BMPs on scleral remodeling, their possible role in choroidal thickness (CHT) was shown to be related to myopia progression [43, 44]. Myopia was induced in young guinea pigs by using a negative magnification rigid gas permeable contact lens. Changes in axial length, CHT, and scleral thickness during myopia induction were measured, and CHT decreased while the axial length increased during the process. Further, gene expression levels of BMP-2 and DNA binding inhibitor 3 (ID3) were down-regulated. The ID3 gene serves as a downstream transcriptional target of BMP signaling and can be used as a reporter of BMP pathway activity [45]. The analysis of gene expression patterns and biometric parameters revealed a significant inverse correlation between CHT and BMP-2 expression levels. These findings provide experimental evidence supporting BMP-2’s role as a negative regulator of myopia progression [44].

Guinea pig studies have established BMPs as key regulators of scleral and choroidal remodeling [37–40, 44], marking BMPs as attractive targets for myopia control. While preliminary data implicate BMP-2 in these processes, comprehensive mechanistic understanding and therapeutic development require further exploration of its precise roles in ocular growth regulation.

### BMPs and strabismus

Strabismus is the inability of both eyes to look at the same point in space at the same time, resulting in impaired binocular synergy [46]. Strabismus affects the accuracy and comfort of the patient’s vision and has a prevalence of about 4 percent of the population [47]. The cause of strabismus is unknown and may be related to neurological abnormalities, central imbalances, and genetics [46]. Treatments include non-surgical and surgical treatments. Surgical treatment at the level of the EOMs is now recognized as an effective form of correction, but the postoperative recurrence rate is relatively high, and it is not easy to obtain completely satisfactory postoperative results [48]. Non-surgical treatments, including monocular masking therapy, medication, and orthodontic training, also fail to provide a long-lasting and complete cure, and pharmacological treatments are prone to cause complications [49]. In the search for more efficient and non-invasive treatments, studies have explored the effects of BMPs on EOMs.

Anderson et al. injected BMP-4 into the superior rectus muscle of the adult rabbits and then evaluated muscle strength and muscle fiber-related indicators. They found that BMP-4 could produce significant and lasting muscle weakening effects on the muscle, including the reduction of muscle power and muscle volume. The muscle-weakening effect persisted for some time until the injection ceased [49]. In addition to the effects of BMP-4 on EOMs, studies in the field of cardiology found that MMPs may affect myocardial contractile force and lead to heart failure [50, 51], suggesting that MMPs may also affect the contractility of EOMs, and the ECM is disrupted in the strabismus patients [52]. This hypothesis was supported by the study on EOMs, in which Lee and Choi determined the expressions of MMPs, TIMPs and BMP-4 in the medial rectus muscle of patients with intermittent exotropia. They found that the levels of MMPs and BMP-4 were positively correlated with deviation angle, age of surgery, and duration of the disease, while TIMPs were inversely correlated. It can be inferred that the high levels of MMP-2 and MMP-9 in the medial rectus muscle weaken its contractility, leading to the contraction force of the lateral rectus muscle being relatively stronger, which may result in exotropia. On the other hand, the researchers speculated that increased MMPs might induce more fibrotic changes postoperatively, which may reduce the contractility of the medial rectus muscle and increase the risk of postoperative recurrence [52].

The use of BMPs to regulate EOM strength provides a new potential therapeutic option for strabismus treatment. The mechanism by which BMP-4 affects EOM changes is not yet clear, and further research is needed to clarify the specific role of BMP-4 in the pathogenesis of strabismus and its safety and effectiveness for the treatment.

### BMPs and corneal diseases

The cornea is a transparent fibrous, avascular tissue, which is involved in the composition of the refractive system. Corneal injury or infection induces opacity and neovascularization (CoNV), frequently culminating in vision-impairing fibrosis and chronic pain [53]. Corneal transplantation remains the gold-standard treatment for vision-impairing scarring. However, its widespread application is constrained by two critical challenges: (1) global donor shortages and (2) variable graft rejection rates that can compromise visual outcomes [54]. Therefore, exploring new effective and safe non-surgical methods to treat corneal scar or corneal fibrosis is necessary.

**Fig. 4:**
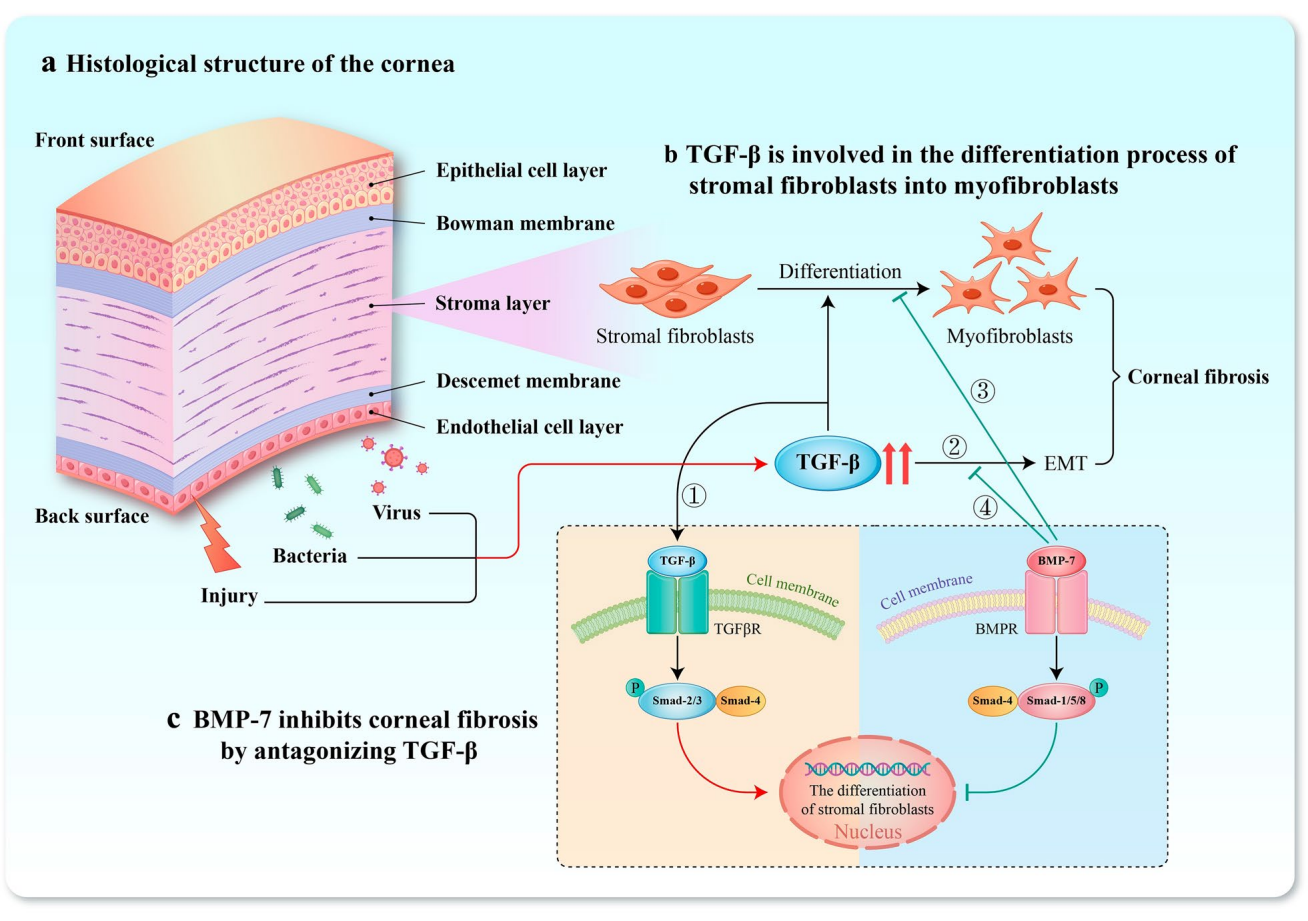
Bone morphogenetic protein (BMP)-7 inhibits corneal fibrosis mainly by antagonizing the effects of transforming growth factor (TGF)-β. **a** The histological structure of the cornea, from anterior to posterior, consists of the epithelial cell layer, the Bowman layer, the stromal layer, the Descemet membrane and the endothelial cell layer. **b** TGF-β is involved in the differentiation process of stromal fibroblasts into myofibroblasts. When the cornea is subjected to trauma, bacterial, or viral infections, the secretion of TGF-β increases. TGF-β participates in the differentiation of stromal fibroblasts through the classical Smad pathway (①). TGF-β can also induce epithelial-mesenchymal transition (EMT) (②). **c** BMP-7 exhibits an antagonistic effect on TGF-β. BMP-7 binds to bone morphogenetic protein receptor (BMPR) and activate Smad1/5/8 phosphorylation and then competitively bind to Smad4 molecules to antagonize the effects of Smad2/3 induced by TGF-β pathway, thereby inhibiting the differentiation of stromal fibroblasts into myofibroblasts (③). BMP-7 can also inhibit TGF-β-induced EMT through the pathway (④)

Previous studies have shown that the conversion of stromal fibroblasts to myofibroblasts is a critical step in the formation of corneal fibrosis [55]. Continued activation of myofibroblasts leads to collagen accumulation and overproduction of ECM, which results in corneal fibrosis and blurred vision [54, 56]. Hence, the modulation of myofibroblast activation emerges as a pivotal therapeutic strategy to control and prevent corneal scarring. Notably, TGF-β, which is secreted by injured corneal cells, and the TGF-β signaling pathways play a crucial role in the transdifferentiation process from stromal fibroblasts into myofibroblasts [57]. As an antagonist of TGF-β, BMP-7 can counteract the effects of TGF-β, and reduce the corneal fibrosis and epithelial-mesenchymal transition (EMT) by activating the Smad1/5/8 signaling pathway and competitively inhibiting Smad2/3 [58, 59] (Fig. 4). Some initial studies have revealed the anti-fibrotic effects of BMP-7 in the cornea and other organs [54, 56, 58–62].

It has been demonstrated that exogenous BMP-7 can reduce renal fibrosis [60], so exogenous BMP-7 might also inhibit tissue fibrosis and inflammation in the cornea after alkali burns [58]. In one study, sodium hydroxide was used to establish a mouse model simulating alkali burns. BMP-7 was delivered into the affected corneal tissues using adenoviral gene transfer. The results demonstrated that BMP-7 by adenoviral gene transfer effectively suppressed the expression of MMP-9 in burned tissues and halted the degradation of the epithelial basement membrane during corneal healing, thereby preserving the integrity of the basement membrane. It also inhibited the expression of type Ia2 collagen genes, reduced myofibroblast expression in the healing stroma, and promoted the proliferation of conjunctival epithelial cells. Underlying mechanisms could have included the attenuation of Smad2/3 signaling along with the upregulation of Smad1/5/8 signaling. Furthermore, BMP-7 could also reduce the number of monocytes and macrophages invading the burned cornea, which may account for the lower expression of inflammatory factors in the burned cornea [58]. Taken together, data suggest that BMP-7 contributed to the healing of the injured cornea and reduced scar formation. Overexpression of BMP-7 may be an effective strategy for the treatment of ocular alkali burns. Apart from adenoviral transfer, nanoparticles can also be used as carriers to deliver BMP-7 to target cells or tissues. For example, polyethylene amine (PEI) conjugated with gold nanoparticles (PEI2-GNP), followed by BMP-7 gene delivery into corneal stromal cells in a rabbit model of laser-induced corneal fibrosis established via excimer laser keratectomy has been demonstrated [61]. The authors found that BMP-7 significantly attenuated the degree of corneal clouding without causing stromal cell apoptosis. The molecular mechanism of the anti-fibrotic effect of BMP-7 in corneas was realized by increasing the expression of inhibitory Smad6 [61].

Mohan et al. further validated the efficacy of anti-corneal fibrosis through the PEI2-GNP-mediated combination therapy of BMP-7 and hepatocyte growth factor (HGF) [54]. The findings showed that HGF elicited apoptosis in human corneal myofibroblasts, whereas it had no such effect on human corneal fibroblasts. BMP-7 and HGF engage in two separate pathways that promote corneal wound healing, with BMP-7 regulating TGF-β signaling and HGF selectively inducing apoptosis in myofibroblasts. The combined treatment was less toxic and restored corneal transparency [54]. Further research should evaluate the translational potential of dual BMP-7/HGF therapy via PEI2-GNP, including long-term outcomes and synergistic mechanisms in corneal wound healing.

Another possible cause of corneal opacity can be attributed to EMT [63]. During EMT, epithelial markers such as E-cadherin, β-catenin and zonular occludens-1 (ZO-1) are downregulated, while interstitial markers such as fibronectin, vimentin and α-smooth muscle actin (α-SMA) are upregulated. It causes the cornea to change from an epithelial phenotype to an interstitial phenotype or a highly motile fibroblast [63]. Based on the anti-corneal fibrosis effect of BMP-7, Chung et al. found that BMP-7 alleviated TGF-β-induced EMT and did not affect epithelial regeneration by detecting the epithelial markers and interstitial markers mentioned above [59].

Histone deacetylase inhibitors have also been hypothesized to play a role in corneal fibrosis because of their antifibrotic effects in pulmonary, cardiac, and renal fibrosis [64–66]. One study explored the effect of the histone deacetylase inhibitor ITF2357 in alleviating corneal fibrosis in vitro and clarified the molecular mechanism by which ITF2357 regulated the TGF-β/Smad/BMP-7 signaling axis and its downstream target ID3 gene thereby exerting an anti-fibrotic effect [56].

Corneal angiogenesis and corneal lymphangiogenesis (CL) subsequent to trauma or infection can also impact the transparency of the cornea [67]. Angiogenesis is defined as the growth of new blood vessels, a process known as “sprouting angiogenesis”. In this process, endothelial cells differentiate into tip cells and stalk cells with distinct morphologies. Tip cells are located at the leading front of the new blood vessels and possess migratory capabilities, guiding the growth of new blood vessels and determining their path. Stalk cells, positioned behind the tip cells, have strong proliferative abilities and form the main body of the new blood vessels, maintaining their stability. Only by maintaining the balance between these two processes can new sprouts be formed [68]. During this process, VEGF will trigger individual endothelial cells to form tip cells, which can induce vascular sprouting and growth. Stalk cells then proliferate, form a lumen and establish blood circulation. A study found that BMP-4 could inhibit CoNV by interfering with the formation of tip cells [69]. After corneal injury, neutrophils produce neutrophil extracellular traps (NETs), which promote neovascularization [70]. The synthesis of NETs not only causes the formation of inflammatory blood vessels but also aggravates corneal edema and tissue injury. NETs induce damage to the apical junctional complexes that maintain corneal barrier function, further promoting CoNV [70]. A recent study by Shen et al. demonstrated the inhibitory effect of BMP-4 on NETs-induced CoNV and apical junctional complexes injury, helping to repair corneal barrier function [71].

An imbalance between proangiogenic and antiangiogenic factors constitutes the principal pathological mechanism underlying CoNV [72]. After corneal damage, various cells, such as epithelial cells and vascular endothelial cells, generate angiogenic factors that facilitate the growth of new blood vessels at the inflammation site [73]. Although corticosteroids, anti-vascular endothelial growth factor (VEGF) preparations and non-steroidal anti-inflammatory drugs are the primary medications for the treatment of CoNV, most of these approaches have side effects that impede the repair of corneal epithelial wounds [69, 74]. Therefore, it is necessary to discover new alternatives to inhibit the formation of CoNV. BMP-4 is one of the key proteins that has become the subject of study in recent years. A few studies have proved that it participates in corneal injury repair by inhibiting CoNV [69, 74]. BMP-4 can down-regulate angiogenic factors and lymphopoietic factors and can inhibit CoNV and CL formation through the Smad pathway [74]. Compared with anti-VEGF drugs, BMP-4 has a protective effect on the corneal epithelium while inhibiting CoNV, and can promote the migration, proliferation and adhesion of corneal epithelial cells, which has more advantages in the repair of corneal injury [74].

Besides the inhibition for CoNV, there have been other studies investigating the effects of BMPs on the differentiation or stratification of the corneal tissue[75–78]. Kamarudin et al. demonstrated that BMP signaling directs the differentiation of human induced pluripotent stem cells (hiPSCs) into corneal epithelial lineages, offering a potential autologous cell source for patients with bilateral limbal stem cell deficiency (LSCD) that circumvents transplant rejection. Their optimized differentiation protocol, combining BMP-4, all-trans retinoic acid (RA), and epidermal growth factor (EGF), successfully generated both corneal epithelial progenitor cells and terminally differentiated epithelial-like cells. EGF is a growth factor that stimulates cell proliferation, differentiation, growth, and migration, and demonstrated to play a synergistic role with BMP-4 in the differentiation of hiPSCs [75]. Lee et al. further supported the conclusion that BMP-4 induced hiPSC differentiation into corneal epithelial progenitor cells and corneal epithelioid cells. On this basis, they also found that the dose and time of BMP-4 affected the direction of hiPSCs differentiation [77]. Additionally, Nguyen et al. systematically evaluated differentiation protocols for converting human Wharton’s jelly-derived mesenchymal stem cells (WJ-MSCs) into corneal epithelial cells in vitro. Their optimized cocktail combining RA, the TGF-β receptor inhibitor SB505124, BMP-4, and EGF, successfully generated corneal epithelial-like cells. This work establishes WJ-MSCs as a promising alternative cell source for LSCD therapy [78]. Another study conducted by Tiwari et al. demonstrated for the first time that BMP-6 promoted corneal epithelium stratification by inhibiting corneal epithelium proliferation and promoting differentiation. The researchers also found that BMP-6 expression was upregulated in pterygium tissue, but further data are needed to support this conclusion [76].

Nowadays, the applications of BMPs in corneal diseases are mainly focused on anti-fibrosis and inhibiting CoNV. However, most of the current studies lack the support of in-depth molecular mechanisms. Future studies are needed to explore the mechanisms, and initial clinical trials can be carried out to ensure their efficacy and safety.

### BMPs and uveal diseases

The uveal is rich in melanin and blood vessels. Uveal melanoma represents the most prevalent primary intraocular malignancy among adults, leading to symptoms such as blurred vision, visual distortion, and loss of visual field [79]. About 50% of uveal melanomas will metastasize, and the survival rate of patients with metastatic tumors is very low. Common treatments for uveal melanoma include radiation therapy and surgical removal of the eye, but radiation therapy can cause optic neuropathy and macular degeneration, and removal of the eye can cause irreversible damage [80]. Therefore, finding a safe and effective treatment for uveal melanoma is an urgent clinical problem.

BMP-7 is an indispensable cytokine for eye development. One study explored the relationship between BMP-7 expression and the tumorigenicity and malignant behavior of uveal melanoma, as well as the effect of BMP-7 overexpression on tumor growth in vivo [81]. The results showed that decreased expression of BMP-7 leads to the progression of uveal melanoma, whereas the tumors overexpressing BMP-7 have relatively limited invasion, usually confined to and around the lens, suggesting BMP-7 may be a novel therapeutic molecule to inhibit the growth of uveal melanoma [81]. In contrast, subsequent studies demonstrated that endogenous BMP signaling promotes melanoma cell invasiveness—an effect effectively suppressed by Noggin. These findings position Noggin as a potential therapeutic candidate for uveal melanoma intervention [82].

Current evidence presents an apparent paradox regarding BMP signaling in uveal melanoma pathogenesis. This dichotomy parallels findings in oral melanoma, where upregulated BMP-2/4/7 expression activates Smad1/5 phosphorylation and subsequent Smad-dependent signaling, driving EMT and cellular invasion [83]. Consistent with these observations, aged animal models of cutaneous melanoma demonstrate BMP-2-mediated tumor cell invasion in vivo [84]. These collective findings underscore the need to elucidate BMP-dependent mechanisms within the tumor microenvironment, characterize subtype-specific signaling outcomes (particularly for BMP-2/4/7), and determine whether Noggin’s therapeutic potential derives from selective inhibition of specific BMP isoforms.

### BMPs and glaucoma

Glaucoma refers to a group of progressive optic neuropathies characterized by atrophy and depression of the optic papilla, visual field defects, and vision loss [85]. Glaucoma is the leading cause of irreversible blindness, with approximately half of all blindness worldwide being angle-closure glaucoma [85, 86]. Pathologically elevated intraocular pressure (IOP) is the primary risk factor for its development, so IOP-lowering therapy is currently the only effective way to stop the progression of glaucoma [86]. Glaucoma usually progresses slowly, and thus patients usually remain asymptomatic until the condition becomes severe. While existing treatments cannot reverse the damage to the visual system caused by glaucoma, early diagnosis and treatment can prevent further progression of the disease.

Zhang et al. identified three key genes, BMP1, DMD, and GEM, which may be potential biomarkers for glaucoma by weighted gene co-expression network analysis, least absolute shrinkage and selection operator, and gene set enrichment analyses [87]. This study may contribute to the development of effective new methods for the diagnosis and treatment of glaucoma.

**Fig. 5:**
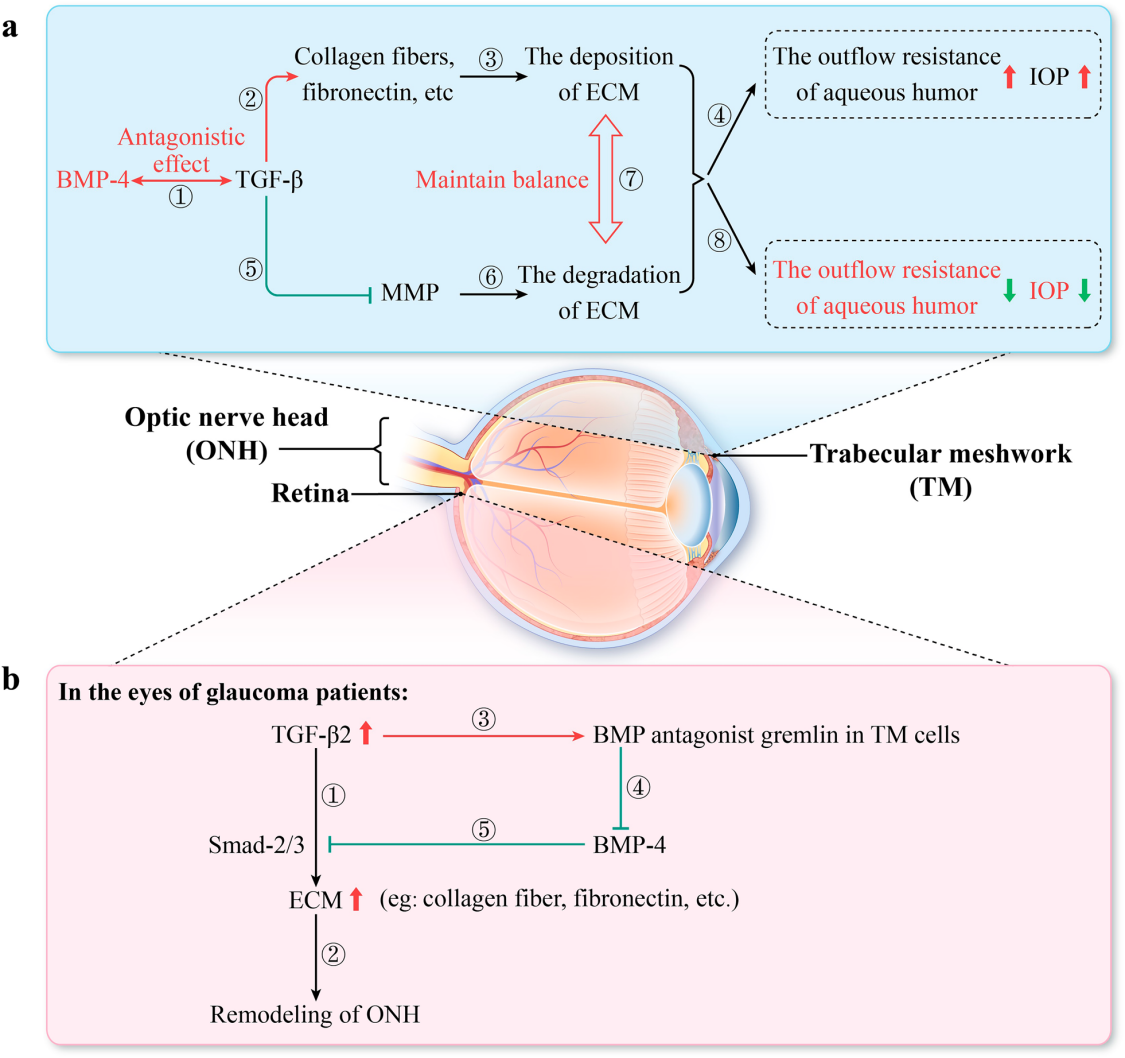
Bone morphogenetic protein (BMP)-4 inhibits the progression of glaucoma by antagonizing transforming growth factor (TGF)-β. **a** BMP-4 can reduce intraocular pressure (IOP) to inhibit the progression of glaucoma. The primary pathogenic factor of primary glaucoma is increased IOP due to elevated outflow resistance of aqueous humor caused by excessive accumulation of extracellular matrix (ECM) in trabecular meshwork (TM) cells. TGF-β promotes the synthesis of ECM proteins (②) to facilitate ECM deposition (③) and inhibits the activity of matrix metalloproteinases (MMP) (⑤) to impede the degradation of ECM proteins (⑥), leading to increased outflow resistance of aqueous humor and elevated IOP (④). BMP-4 exhibits an antagonistic effect on TGF-β (①), blocking the influence of TGF-β on ECM-related proteins in TM cells. The antagonistic effect between BMP-4 and TGF-β helps maintain the dynamic balance between ECM deposition and degradation (⑦), contributing to reduced outflow resistance of aqueous humor and normal IOP. **b** TGF-β2, BMP-4 and gremlin form an autocrine loop. In glaucoma patients, elevated levels of TGF-β2 in the aqueous humor can activate the Smad pathway through Smad2/3 signaling molecules (①), resulting in increased synthesis of ECM proteins and remodeling of the optic nerve head (ONH) (②). Increased levels of TGF-β2 upregulate the expression of the BMP antagonist gremlin in TM cells (③). Gremlin can directly bind to BMP-4, inhibiting its biological activity (④). Therefore, the ability of BMP-4 to antagonize TGF-β2 is suppressed (⑤)

The main etiological factor in primary open-angle glaucoma is elevated IOP due to increased aqueous humor outflow resistance, which is associated with morphological and biochemical changes in the TM [88]. Patients with glaucoma have an accumulation of large amounts of ECM in the TM, the change that may result from a disruption of the balance between ECM deposition and degradation [88]. Previous studies have demonstrated that human TM cells express mRNA for BMPs, BMPRs, and selective BMP antagonists [15]. Several studies have confirmed that BMP-4 and BMP-7 inhibit TGF-β2-induced ECM protein in TM cells, thereby reducing the outflow resistance of aqueous humor [12, 89, 90]. Wordinger et al. confirmed that TM cells can secrete BMP-4, which can block the effect of TGF-β2 on ECM-related proteins in TM cells in a dose-dependent manner [89]. Therefore, BMP-4 plays an important role in maintaining normal TM function by antagonizing the action of TGF-β2. Moreover, this study found that gremlin, a BMP antagonist, inhibited BMP-4 activity and increased aqueous humor outflow resistance by enhancing TGF-β2 action indirectly to increase ECM deposition in TM cells [89] (Fig. 5). Another report demonstrated that antagonism between BMP-4 and TGF-β2 may help to maintain the normal dynamic balance of ECM synthesis and deposition in ONH astrocytes and sieve plate cells[90]. They found that TGF-β2, BMP-4 and gremlin formed an autocrine loop (Fig. 5). Specifically, elevated TGF-β2 expression induces gremlin expression, which in turn blocks the regulation of BMP, leading to increased ECM synthesis and remodeling of ONH [90].

Except for the effects of regulating TM cells, the protective effect of BMP-4 on retinal ganglion cells (RGCs) and axonal regeneration has been verified by observing RGCs’ survival in an experimental glaucoma mouse model [91–93]. It is suggested that the BMP4/Smad1/5/8 pathway may be related to the regeneration of RGCs, though this needs further confirmation [91–93].

### BMPs and cataract

Cataract is one of the leading causes of blindness, and millions of people worldwide suffer from blurred vision caused by cataract [94]. EMT in lens epithelial cells is an important pathologic mechanism in cataract. TGF-β and BMP-7 have different functions in the EMT: TGF-β is a potent inducer of EMT, but BMP-7 counteracts the pro-fibrotic effects of TGF-β [95, 96].

In 2017, researchers investigated for the first time the role of exogenous BMP-7 in regulating TGF-β-induced intralens EMT. They administered both TGF-β2 alone and a combination of TGF-β2 with BMP-7 to rat lens epithelial cells, and the results showed BMP-7 could block TGF-β2-induced EMT in lens epithelial cells in a dose-dependent manner, suggesting BMP-7 is expected to be a therapeutic agent for anterior subcapsular cataract (ASC) and posterior capsular opacification [97].

Although exogenous BMPs can antagonize TGF-β-induced EMT, they are complex, unstable, and expensive to produce [98]. Consequently, researchers have developed cost-effective alternatives to recombinant BMPs, including synthetic small-molecule BMP signaling agonists. Investigations comparing BMP-4 and the BMP agonist ventromorphin revealed differential efficacy in blocking TGF-β-induced lens epithelial cell transdifferentiation: while BMP-4 successfully inhibited TGF-β-mediated EMT, ventromorphin demonstrated no significant inhibitory effect [99]. Wu et al. further elucidated the roles of BMP-4 and BMP-7 in cataract pathogenesis and their possible mechanisms. They confirmed the Notch pathway as a potential mechanism for the inhibition of EMT by BMP-4 and BMP-7 in vivo. BMP-4 and BMP-7 induce cell cycle arrest by modulating the Notch signaling pathway, thereby inhibiting lens epithelial cell proliferation and TGF-β2-induced lens epithelial cell migration and EMT[100]. These studies demonstrate that BMP-4 and BMP-7 are expected to be effective agents for preventing cataract.

Although these studies highlight the therapeutic promise of BMP-4 and BMP-7 for cataract prevention, key limitations persist. The findings currently lack in vivo validation in mammalian models, and the mechanistic relationship between TGF-β inhibition and BMP-Smad pathway activation in mediating these protective effects remains poorly characterized.

### BMPs and fundus diseases

#### Retinal injury

Müller glial cells, the major glial cells in the retina, are mitotically quiescent under normal conditions but are stimulated to proliferate under certain pathological states [101]. These glial cells play dichotomous roles after injury: they secrete neuroprotective factors to sustain surviving neurons but also form a glial scar that physically and molecularly inhibits axonal regeneration [102]. It has been demonstrated in avian retinas that Müller glial cells respond to neurotoxic injury by proliferating and dedifferentiating into neural progenitor-like cells. Fischer et al. tested the ability of EGF, BMP-4, and ciliary neurotrophic factor (CNTF) to regulate the proliferation and dedifferentiation of Müller glial cells into neural progenitor-like cells in response to the retinal injury. The three cytokines significantly reduced the proliferation of Müller cells after the injury, and they had a protective effect against N-methyl-D-aspartic acid-mediated excitotoxicity in retinal neurons. Unlike BMP-4, the effect of CNTF on glial proliferation was independent and its neuroprotective effect was long-term while BMP-4 was short-term [102]. Although BMP-4 has been confirmed to have protective effects on retinal damage, there is a lack of understanding of the role of BMPs in the proliferation of Müller glial cells. To further investigate the signaling pathway required for BMP-mediated Müller glial cell proliferation, Ueki and Reh found that the BMP/Smad1/5/8 signaling was essential for the proliferation of Müller glial cells, and EGF-induced proliferation of Müller glial cells was also partly mediated by BMP/Smad1/5/8 signaling [103]. The findings of these studies may provide new strategies for the treatment of retinal injuries.

#### Retinoblastoma (Rb)

Rb is the most common intraocular malignant tumor in children, which causes a serious threat to children’s vision and lives [104]. Multiple studies on human myeloma cell lines have shown that human myeloma cell lines lack bone morphogenetic protein receptor I (BMPR-I, one of the three BMP receptors, including BMPR-IA and BMPR-IB, distributed in human ocular tissues) [105]. These cell lines are human Rb cell lines that are isolated from unilateral and bilateral tumors. Therefore, it is reasonable to assume that Rb cells also lack BMPRs. Unlike human myeloma cell lines, all BMPR subtypes have been shown to be expressed in multiple Rb cell lines, including BMPR-IA, BMPR-IB, and BMPR-II [106]. Exogenous BMP-4 was found to promote apoptosis of Wills Eye Research Institute-Rb-1 cells but had no effect on cell proliferation [106]. Another study showed that BMP-4 in conjunction with RA promoted cell apoptosis to a greater extent than that using BMP-4 or RA alone [107]. These studies inferred that the mechanism of BMP-4 promoting apoptosis involved Smad1/5/8 signaling molecules [106, 107], but the role of BMP-4 in Rb cells is complex, involving multiple signaling pathways and mechanisms. Further studies should be carried out to illustrate the mechanisms, and BMP-4 is expected to provide a new strategy for the treatment of Rb.

#### Diabetic retinopathy (DR)

DR is the most common retinal vascular disease and is the leading cause of vision loss in the elderly [108]. The visual loss caused by DR is mainly due to the disruption of the blood-retinal barrier (BRB), which leads to macular edema, retinal detachment and vitreous hemorrhage [108]. The retinal barrier includes the inner blood-retinal barrier (iBRB) and outer blood-retinal barrier (oBRB). Inner BRB is mainly composed of retinal capillary endothelial cells and their close junctions, oBRB is mainly composed of RPE cells and their junctions. Together, iBRB and oBRB maintain the stability of the retinal environment and normal visual function [109]. Studies have found BMPs involved in the pathogenesis of DR.

Researchers from Al Shabrawey’s team focused on the distribution of BMP-2 and BMP-4 in the eyes of DR patients and their effects on the BRB [110, 111]. The expression levels of BMP-2 and BMP-4 in the retina of diabetic patients are higher than those of normal people, which may be related to the pathogenesis of DR [110–112]. BMP-2 can affect the function of iBRB by enhancing leukocyte adhesion and by inducing pro-inflammatory cytokines secretion [110]. BMP-4 also mediates upregulations of MMP-2 and MMP-9 in DR, which can cause the degradation of retinal capillary basement membrane components and tight junction proteins between retinal capillary endothelial cells, thereby increasing BRB permeability [111]. In addition to upregulating MMP activity, BMP-4 disrupts the tight junction protein ZO-1 and activates both the canonical Smad signaling pathway and the non-canonical p38 MAPK pathway in human retinal endothelial cells (HRECs) [111]. Moreover, Vogt et al. confirms that BMP-4 may play a role in DR-related ocular angiogenesis by stimulating the release of VEGF from RPE cells [113]. Collectively, these findings implicate BMPs in the pathogenesis of DR and highlight their potential as therapeutic targets for future DR interventions.

#### Age-related macular degeneration (AMD)

AMD is the most common cause of vision impairment in people over the age of 55 in the developed world [114]. The pathology of AMD is characterized by degenerative atrophy of the outer layer of the retina, including photoreceptor, RPE, Bruch’s membrane, and the choroidal capillaries [114–117]. The RPE secretes many growth factors to maintain normal function of the retina and choroid, and both early and late AMD lesions are mainly targeted at RPE. RPE dysfunction is thought to play a key role in the pathogenesis of AMD [118]. Currently, the primary medications used for treating AMD are anti-VEGF drugs. Anti-VEGF drugs are administered through intravitreal injection, which carries the risk of causing increased IOP, intraocular inflammation, retinal detachment, ocular hemorrhage, and systemic diseases after injection [119, 120]. To ensure therapeutic efficacy, long-term follow-up and treatment are necessary, but long-term treatment imposes a significant financial burden on patients [114–117]. Therefore, new therapeutic targets need to be identified to modulate angiogenesis in patients with AMD.

**Fig. 6:**
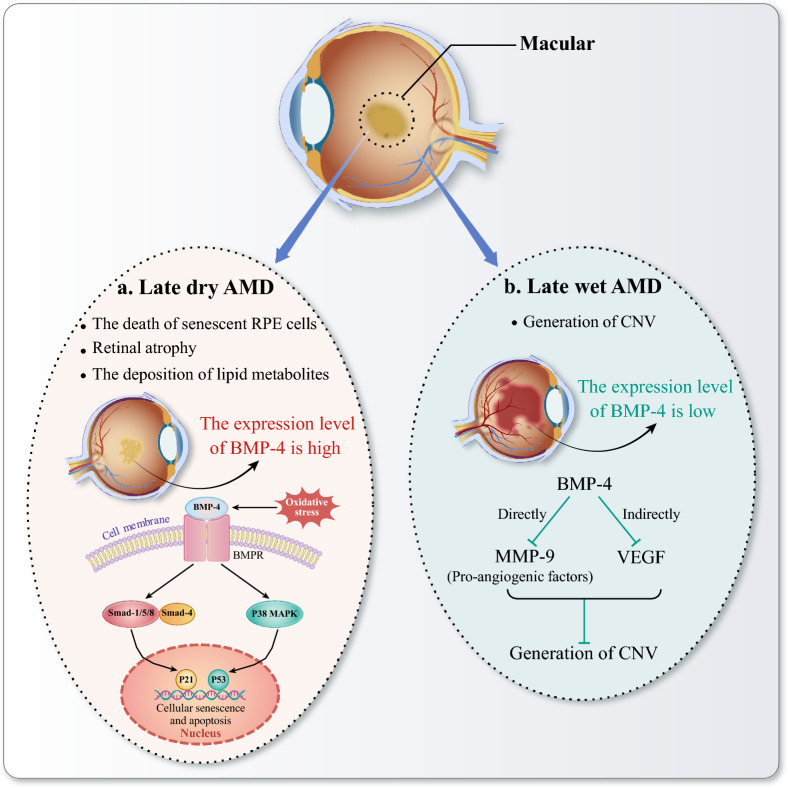
Bone morphogenetic protein (BMP)-4 might be a new therapeutic target for age-related macular degeneration (AMD). BMP-4 exhibits differential expression in the macular tissues of patients with dry and wet AMD. **a** In late dry AMD, the death of aged retinal pigment epithelium (RPE) and the deposition of lipid metabolites both contribute to geographic atrophy of the retina. BMP-4 is highly expressed in patients with advanced dry AMD. In response to external stimuli such as oxidative stress, BMP-4 activates the Smad pathway and the p38 MAPK pathway to mediate AMD. p21 and p53 are crucial factors that induce cell cycle arrest. The Smad1/5/8 and Smad4 complex activate p21, while the p38 MAPK pathway activates downstream p53 molecule, thereby inducing cellular senescence and apoptosis. **b** Late wet AMD is primarily associated with the generation of choroidal neovascularization (CNV). BMP-4 is lowly expressed in patients with advanced wet AMD. BMP-4 inhibits the formation of CNV by directly suppressing the angiogenesis-promoting factor matrix metalloproteinase-9 (MMP-9) and indirectly inhibiting vascular endothelial growth factor (VEGF)

It has been observed that BMP-4 exhibits differential expression patterns in the macular of individuals suffering from dry and wet AMD [121]. Specifically, BMP-4 is notably overexpressed in the RPEs of patients with advanced stages of dry AMD, whereas it is significantly under-expressed in those with wet AMD [121] (Fig. 6). Long-term oxidative stress is also thought to play an important role in the pathogenesis of dry AMD [122], which will induce cellular senescence leading to RPE atrophy and blindness [115]. Considering BMP-4 is significantly expressed in the RPE and Bruch’s membrane of AMD patients, the researchers found that the oxidant-induced senescence of RPE cells is mediated by BMP-4 through the Smad and p38 signaling pathways [123, 124]. Therefore, BMP-4 might be a new therapeutic target for inhibiting oxidative stress and aging effects in AMD.

In addition to dry AMD, a study evaluated the expression of BMP-4 on mouse models of CNV, and confirmed that the level of tumor necrosis factor (TNF) was negatively correlated with the level of BMP-4, and TNF downregulates the expression of BMP-4 in the RPE [121]. The researchers also found that the c-Jun N-terminal kinase (JNK) signaling pathway was involved in this regulatory process and that the Sp1 transcription factor, as a downstream target of this signaling pathway, could be a new approach to the treatment of wet AMD [121]. Furthermore, Xu et al. confirmed the anti-angiogenic effect of BMP-4 in laser-induced CNV model and illustrated that the mechanism of BMP-4 regulation of angiogenesis was achieved through direct inhibition of MMP-9 and indirect inhibition of VEGF [125]. Activin receptor-like kinase 1(Alk1) is known to be a receptor for BMP-9, and Ntumba et al. explored the role of the BMP-9/AlK1 axis in CNV using an aged mouse model of macular degeneration. The experimental results suggested that BMP-9 could inhibit CNV in wet AMD and enhance the efficacy of anti-VEGF medications [126]. Meanwhile, they found that the combination of BMP-9 and anti-VEGF drugs could alleviate the possible adverse effects of anti-VEGF alone and may serve as a new direction for the treatment of AMD in the future [126].

#### Proliferative vitreoretinopathy (PVR)

The junction between the neuroepithelial layer of the retina and the RPE layer lacks tightness, constituting the histological foundation for retinal detachment [127]. Rhegmatogenous retinal detachment is the most common type, which occurs on the basis of the formation of a retinal tear through which the liquefied vitreous body enters under the neuroepithelial layer, separating the RPE layer from the neuroepithelial layer [127]. PVR is a severe complication after rhegmatogenous retinal detachment. Current studies found crucial cells in the development of PVR are RPE cells, glial cells, fibroblasts and macrophages [128]. The researchers investigated the antifibrotic effects of BMP-7 in an in vivo and in vitro model of PVR. BMP-7 was expressed in the retinal precursor of patients with PVR, and its expression was downregulated during the development of PVR. BMP-7 strongly inhibited the development of PVR and suppressed the phenotype of RPE and TGF-β2-induced EMT [129]. Thus, BMP-7 may be a promising molecule for the treatment of PVR.

### BMPs and other ocular diseases

#### Graves’ ophthalmopathy (GO)

GO, an ocular manifestation of thyroid dysfunction, is seen in 25%–50% of patients with Graves’ disease [130]. GO has a high incidence, is characterized by prominent swelling of the eye, eye movement disorders, diplopia, and exposure keratitis, and in severe cases, damage to the optic nerve can lead to vision loss [130]. The research on the pathogenesis of GO indicates that orbital fibroblasts are one of the main cells causing orbital muscle enlargement and inflammation. The pro-inflammatory cytokines produced by orbital fibroblasts can lead to the production of collagen fibers and GAGs, thereby causing ocular soft tissue edema and fibrosis [130, 131]. Steroidal therapy is the first-line treatment option for GO, but the efficacy is limited. There is a need to investigate new therapies to inhibit the development of GO and to gain a deeper understanding of the underlying pathogenesis.

It was concluded that BMP-7 transcript levels were downregulated in Graves’ orbital tissue, suggesting that it may be involved in the pathogenesis of GO [132]. Studies have investigated the involvement of exogenous recombinant human BMP (rhBMP)-7 on the pathogenesis of GO and assessed its therapeutic effects [132]. Exogenous BMP-7 inhibited TGF-β-induced fibrinogen production and pro-inflammatory cellular mediators. Additionally, BMP-7 promoted the activation of the anti-fibrotic signaling molecule Smad1/5/8, while inhibiting the pro-fibrotic signaling molecule Smad2/3 and the pro-inflammatory signaling molecules NF-κB and Akt proteins [132]. The investigation of signaling molecules provides the molecular basis for BMP-7 as a new potential therapeutic agent.

#### Herpes simplex keratitis (HSK)

Herpes simplex virus type-1 (HSV-1) primarily infects mucous epithelial tissues of the eye and the oral face, after which the virus reaches the trigeminal ganglion and remains latent in nerve cells for long periods [133, 134]. HSV infection of the eye can occur at any location, and HSK is the most common one which can cause corneal opacity and vision loss [134]. Latency-associated transcript (LAT) is a viral gene detected during the incubation period of HSV-1 and is also the only viral gene that is transcribed in large quantities during this period [133]. Previous studies have proposed the hypothesis that BMPs may interact with LAT. Hamza and colleagues conducted an investigation into the impact of HSV-1 LAT on the expression of substance P which is a neuropeptide that plays a role in neurogenic inflammation, immune response modulation, and pain sensation, in cultured embryonic trigeminal nerve cells derived from rats [135]. The findings indicated that BMP-7 reduced substance P immunoreactivity in a dose-dependent manner, while the expression of LAT in cells enhanced the expression of substance P [135]. BMP-7 affects substance P expression providing the possibility that BMP-7 attenuates HSV-1 infection reactivation.

## Discussion

As multifunctional cytokines, BMPs have been gaining increasing attention for treating ocular diseases. The studies mentioned in this review mainly focus on the therapeutic effects of BMPs and their molecular mechanisms involved in the treatment or pathogenesis over the past two decades.

BMPs are important for the normal function and morphology of the mammalian eye and are involved in the development and differentiation of the embryonic ocular tissues [15, 19, 21–23, 136–138]. BMP-2, BMP-4, and BMP-7 are the main subtypes of BMPs widely encountered in ophthalmology [11]. BMP-2 is involved in the scleral remodeling process [37–41, 44], and BMP-4 is associated with the onset and treatment of glaucoma and strabismus [12, 49, 52, 89–91, 93]. In addition, BMPs can promote the repair and regeneration of corneal and retinal tissue and inhibit the EMT of cells from the cornea and lens [56, 139]. BMP-4 and BMP-7 mainly play roles in corneal fibrosis and CoNV, not only antagonizing the TGF-β-induced EMT of keratocyte, but also slowing down the progression of CoNV [54, 56, 58, 59, 61, 69, 71, 74, 140, 141]. However, the effect of BMPs on regulating the formation of new blood vessels in the retina and choroid still remains controversial. Vogt et al. believed that BMP-4 induced VEGF and promoted the formation of new retinal blood vessels [113], while Xu et al. found that BMP-4 inhibited fundus angiogenesis [125]. The effect of BMPs on neovascularization in fundus diseases still needs further exploration.

There have been some contradictory findings shown in the studies of treating certain ocular diseases with BMPs, especially in uveal melanoma [83, 84]. We speculate that the effects of these contradictions are caused by the different subtypes of BMP that different researchers used in their studies. As mentioned before, BMP-2 may induce the EMT process by activating the Smad signaling pathway, enhancing the invasion and migration abilities of cells, and thereby mediating the invasion of the tumors. On the other hand, BMP-7 may limit the invasion range of the tumor by antagonizing the fibrosis of the TGF-β signal and EMT [83]. Additionally, the different microenvironments of the tumor cells may also be one of the possibilities leading to the contradictory effects of BMPs in uveal melanoma [142]. Similarly, BMPs also play a contradictory role in the formation of fundus blood vessels. They can both promote and likewise inhibit angiogenesis [111, 125]. In DR, BMP-4 can promote the permeability of the BRB by enhancing the activities of MMP-2 and MMP-9, activating the Smad and MAPK-p38 signaling pathway, thereby promoting angiogenesis [111]. On the other hand, BMP-4 inhibits angiogenesis by suppressing the activities of MMP-9 and VEGF in AMD [125]. This might be due to the complex interactions between BMPs and different signaling pathways, which can have different effects in different disease contexts. Regarding the diverse roles of BMPs in diseases, well-designed studies are needed to clarify their roles and interactions in the future.

**Fig. 7:**
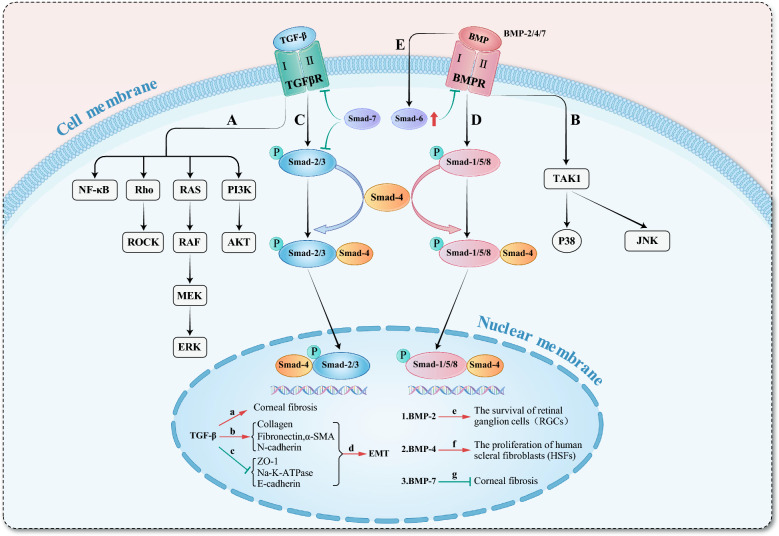
Classical and non-classical signaling pathways of transforming growth factor (TGF)-β and bone morphogenetic protein (BMP). **A** Non-classical signaling pathways of TGF-β, including the nuclear factor-kappa B (NF-κB) pathway, Ras homolog gene family-guanosine triphosphatases (Rho-GTPase) pathway and its downstream Rho-associated coiled-coil containing protein kinase (ROCK) branch, rat sarcoma (RAS)–rapidly accelerated fibrosarcoma (RAF)–mitogen-activated extracellular signal-regulated kinase (MEK)–extracellular signal-regulated kinase (ERK) pathway, and phosphatidylinositol 3-kinase/protein kinase B (PI3K/AKT) pathway. **B** Non-classical signaling pathways of BMP, such as the p38 mitogen-activated protein kinase (p38 MAPK) and c-Jun N-terminal kinase (JNK) branches activated via the TGF-β activated kinase 1 (TAK1) pathway, are categorized under the mitogen-activated protein kinase (MAPK) signaling cascade. **C** Classical Smad signaling pathway of TGF-β. TGF-β can activate this pathway after binding to transforming growth factor β receptor (TGFβR), induce corneal fibrosis (a), and promotes the expression of collagen, fibronectin, α-smooth muscle actin (α-SMA) and N-cadherin (b); while inhibiting the expression of zonular occludens-1 (ZO-1), Na–K-ATPase, and E-cadherin (c), thereby inducing epithelial mesenchymal transition (EMT) (d). **D** Classical Smad signaling pathway of BMP binding to bone morphogenetic protein receptors (BMPR). BMP-2 aids in the survival of retinal ganglion cells (e), BMP-4 promotes the proliferation of human scleral fibroblasts (HSFs) (f), and BMP-7 inhibits corneal fibrosis (g). The pathways depicted in panels (**C**) and (**D)** illustrate the antagonistic effect between TGF-β and BMP, which arises from their competitive binding to the Smad4 molecule. **E** The Smad6 molecule primarily exerts inhibitory effects. BMPs can increase the expression level of Smad6, and in turn, the inhibitory Smad6 molecule inhibits the actions of BMPs

Researches have confirmed the involvement of the classical TGF-β/Smad signaling pathway and the BMP/Smad signaling pathway in the pathogenesis or therapeutic processes of the ocular diseases, encompassing corneal fibrosis, glaucoma, AMD, amongst others [56, 58, 59, 91–93, 103, 123, 124, 143]. The antagonistic effects of TGF-β and BMP are primarily mediated through their competitive binding to the Smad4 signaling molecule (Fig. 7). Consequently, TGF-β is a protein that necessitates attention when investigating the mechanisms of action of BMPs. The antagonistic effects between TGF-β and BMP manifest in various pathological processes, such as EMT, ECM deposition and degradation, neovascularization and lymphangiogenesis, the survival of RGCs and BRB permeability [56, 58, 59, 74, 103, 111]. Based on these observations, we speculate that the TGF-β/Smad pathway and the BMP/Smad pathway may also participate in the pathogenesis of scleral remodeling in myopia, EMT in the lens during cataract progression, proliferation of Müller glia cells following retinal injury and neovascularization in AMD. Therefore, further investigations are needed to illustrate these mechanisms.

BMPs have been shown to be involved in the pathogenesis of many ocular diseases, so it may become a new therapeutic approach. Currently, there are limited therapeutic data on the long-term efficacy and safety of BMPs in ocular diseases, and further clinical trials are needed. Although natural BMPs can be extracted from bones, they are prone to rapid degradation, and are costly to extract [144]. Future research should be more inclined towards the application of rhBMPs. Compared to natural BMPs, the therapeutic dose of rhBMPs is much higher than the physiological dose, and the high concentration of rhBMPs may also increase the probability of inflammation and edema [144]. Moreover, it has a short half-life and requires repeated injections, further increasing the financial burden on patients [144]. Besides the origins of BMPs can affect their therapeutic effects, the delivery means also determines their efficacy. Thus, there is a need to provide appropriate carriers or delivery systems for delivery BMPs to exert their effects thoroughly [98]. The carriers for delivering BMPs should be non-toxic, anti-degradation, biocompatible, and possess low incidence of inflammatory responses, high targeting ability, and have sites that can bind to target cells or tissues [98, 144, 145]. Current research uses carriers such as collagen, synthetic polymers, hydrogels, and nanoparticles, but none of them fully meet all the above requirements [145]. Future studies should continue to focus on exploring the carriers and the optimal effective dosage of BMPs to treat ocular diseases.

Overall, additional foundational studies are necessary to substantiate the therapeutic potential of BMPs. Moreover, indepth investigations into their molecular mechanisms are essential to facilitate the translation of preclinical findings into clinical trials and, ultimately, their application in the treatment of ocular diseases.

## Conclusion

The exploration of BMPs as therapeutic agents for ocular diseases represents a promising and emerging field. However, their efficacy and mechanisms of action in humans remain to be fully elucidated. Future studies should focus on the application of BMPs and their inhibitors in ocular pathologies, with particular attention to the underlying regulatory signaling pathways and optimal delivery strategies. Furthermore, careful consideration must be given to the translational potential of preclinical findings, ensuring a rigorous and effective progression from animal models to clinical trials.

## Data Availability

Not applicable.

## Abbreviations

Alk1: Activin receptor-like kinase 1
AMD: Age-related macular degeneration
ASC: Anterior subcapsular cataract
BMPs: Bone morphogenetic proteins
BMPR: Bone morphogenetic protein receptor
BMPR-IA: Bone morphogenetic protein receptor IA
BMPR-IB: Bone morphogenetic protein receptor IB
BMPR-II: Bone morphogenetic protein receptor II
BRB: Blood-retinal barrier
CAM: Cartilage-associated matrix
CHT: Choroidal thickness
CL: Corneal lymphangiogenesis
CNTF: Ciliary neurotrophic factor
CNV: Choroidal neovascularization
CoNV: Corneal neovascularization
DR: Diabetic retinopathy
ECM: Extracellular matrix
EGF: Epidermal growth factor
EMT: Epithelial-mesenchymal transition
EOM: Extraocular muscle
GAGs: Glycosaminoglycans
GO: Graves’ ophthalmopathy
HGF: Hepatocyte growth factor
hiPSC: Human induced pluripotent stem cells
HRECs: Human retinal endothelial cells
HSFs: Human scleral fibroblasts
HSK: Herpes simplex keratitis
HSV: Herpes simplex virus
iBRB: Inner blood-retinal barrier
ID3: DNA binding inhibitor 3
IOP: Intraocular pressure
LAT: Latency-associated transcript
LSCD: Limbal stem cell deficiency
MMPs: Matrix metalloproteinases
NETs: Neutrophil extracellular traps
oBRB: Outer blood-retinal barrier
ONH: Optic nerve head
PEI: Polyethylene amine
PEI2-GNP: Polyethylene amine coupled with gold nanoparticles
PVR: Proliferative vitreoretinopathy
RA: Retinoic acid
Rb: Retinoblastoma
RGCs: Retinal ganglion cells
rhBMPs: Recombinant human BMPs
RPE: Retinal pigment epithelium
TGF-β: Transforming growth factor β
TIMP: Tissue inhibitor of metalloproteinase
TM: Trabecular meshwork
TNF: Tumor necrosis factor
WJ-MSCs: Wharton’s jelly-derived mesenchymal stem cells
ZO-1: Zonular occludens-1
α-SMA: α-Smooth muscle actin
